# A Comparison of Structural Variant Calling from Short-Read and Nanopore-Based Whole-Genome Sequencing Using Optical Genome Mapping as a Benchmark

**DOI:** 10.3390/genes15070925

**Published:** 2024-07-16

**Authors:** Yang Pei, Melanie Tanguy, Adam Giess, Abhijit Dixit, Louise C. Wilson, Richard J. Gibbons, Stephen R. F. Twigg, Greg Elgar, Andrew O. M. Wilkie

**Affiliations:** 1 Clinical Genetics Group, MRC Weatherall Institute of Molecular Medicine, University of Oxford, Oxford OX3 9DS, UK; yang.pei@imm.ox.ac.uk (Y.P.); stephen.twigg@imm.ox.ac.uk (S.R.F.T.); 2 Genomics England Limited, One Canada Square, London E14 5AB, UK; 3 Clinical Genetics Service, Nottingham University Hospitals NHS Foundation Trust, City Hospital, Nottingham NG5 1PB, UK; 4 North East Thames Regional Genetics Service, Great Ormond Street Hospital for Children NHS Foundation Trust, Great Ormond Street Hospital, London WC1N 3JH, UK; 5 MRC Molecular Haematology Unit, MRC Weatherall Institute of Molecular Medicine, University of Oxford, Oxford OX3 9DS, UK

**Keywords:** Bionano optical genome mapping, Oxford Nanopore Technologies, Illumina sequencing, short read, long read, structural variant, copy number variant

## Abstract

The identification of structural variants (SVs) in genomic data represents an ongoing challenge because of difficulties in reliable SV calling leading to reduced sensitivity and specificity. We prepared high-quality DNA from 9 parent–child trios, who had previously undergone short-read whole-genome sequencing (Illumina platform) as part of the Genomics England 100,000 Genomes Project. We reanalysed the genomes using both Bionano optical genome mapping (OGM; 8 probands and one trio) and Nanopore long-read sequencing (Oxford Nanopore Technologies [ONT] platform; all samples). To establish a “truth” dataset, we asked whether rare proband SV calls (*n* = 234) made by the Bionano Access (version 1.6.1)/Solve software (version 3.6.1_11162020) could be verified by individual visualisation using the Integrative Genomics Viewer with either or both of the Illumina and ONT raw sequence. Of these, 222 calls were verified, indicating that Bionano OGM calls have high precision (positive predictive value 95%). We then asked what proportion of the 222 true Bionano SVs had been identified by SV callers in the other two datasets. In the Illumina dataset, sensitivity varied according to variant type, being high for deletions (115/134; 86%) but poor for insertions (13/58; 22%). In the ONT dataset, sensitivity was generally poor using the original Sniffles variant caller (48% overall) but improved substantially with use of Sniffles2 (36/40; 90% and 17/23; 74% for deletions and insertions, respectively). In summary, we show that the precision of OGM is very high. In addition, when applying the Sniffles2 caller, the sensitivity of SV calling using ONT long-read sequence data outperforms Illumina sequencing for most SV types.

## 1. Introduction

Whole-genome sequencing (WGS) is increasingly implemented routinely in clinical settings to identify causative variants in human genetic disease [1,2,3]. As methods to catalogue single nucleotide variants (SNVs) have become more reliable [4,5,6,7,8], attention has turned to improving the calling of structural variants (SVs). Such SVs, defined as variants >50 bp in size [9] (including copy-number variants [CNVs] made up of deletions and duplications and other events comprising inversions, translocations, and complex rearrangements) are inherently more challenging to detect and call in short-read (SR) sequences, owing to both technological and computational limitations. For instance, Illumina platform-based SR WGS typically generates paired-end reads of approximately 300 bp in length, which may struggle to accurately sequence through repetitive regions. Importantly, these repetitive elements, such as *Alu* and *L1* repeats, often play a crucial role in SV mutagenesis through DNA repair and replicative mechanisms [10].

In clinical practice, various other technologies, including array-based technologies and fluorescence in situ hybridization (FISH), are conventionally employed to detect some of the most apparent SVs, involving rearrangements affecting >2–5 Mb of DNA [11,12,13]. More recently, long-range genomic technologies, such as long-read (LR) sequencing and optical genome mapping (OGM), have progressed into some clinical applications, particularly in infectious disease and cancer [14,15,16]. In rare congenital diseases, these novel technologies may also have the potential to improve diagnostic rates and clinical outcomes [17]. With longer read length, LR technologies offer a more comprehensive understanding of challenging regions of the genome, facilitating the identification of causative SVs in patients yet to receive a molecular diagnosis. 

In this work, we aimed to evaluate the clinical performance of three genomic technologies, Illumina SR-WGS, Oxford Nanopore Technologies (ONT) LR-WGS, and Bionano OGM, in detecting rare SVs of potential clinical relevance. Our investigation focused on a model disease cohort affected with a highly clinically and genetically heterogeneous condition, namely, craniosynostosis (CRS) [18]. As part of the 100,000 Genomes Project (100 kGP), 114 CRS families without a genetic diagnosis were initially recruited and sequenced using Illumina SR-WGS technology. Despite intense research scrutiny looking for both causative single nucleotide polymorphisms (SNPs) and SVs, 78 families remained without a molecular diagnosis [7]. From these families, we selected 9 trios comprising an affected child with no genetic diagnosis and two unaffected parents for whom we had access to fresh blood samples for high molecular weight DNA extraction from each family member. By combining analysis using LR ONT WGS and Bionano OGM, we aimed to identify SVs previously overlooked by Illumina WGS. Here, we focus on a set of potentially clinically relevant rare SVs detected using Bionano OGM to identify a “truth dataset” of SVs, which we could use to benchmark the performance of currently used Illumina and ONT variant callers to assess clinical utility in rare disease. 

## 2. Materials and Methods

### 2.1. Ethics

Please refer to Institutional Review Board and Informed Consent statements at the end of the article.

### 2.2. Genome Sequencing

Illumina SR WGS data were generated as part of the 100 kGP as described in Smedley et al. (2021) [19,20]. Data (Data Release V7, 25 July 2019) were accessed in the Genomics England Research Environment (RE) as part of the research study Molecular Genetics of Craniosynostosis (Research Registry project 65); approval was obtained for the export of all data and figures provided in this work. Methods for SV detection in Manta [21] and Canvas [22] files provided for each sequence in the RE were reported in Hyder et al. (2021) [7]. Lumpy SV [23] calling was performed using LUMPYExpress (Lumpy v.0.2.13) as described in https://github.com/arq5x/lumpy-sv (last accessed on 28 May 2024). All data presented were mapped to the GRCh38 reference. In one of the 9 families selected for this study (Case 3), a causative pathogenic variant (*de novo* c.430dup in *HIST1H1E*) was subsequently independently identified by reanalysing previously filtered-out Illumina calls.

### 2.3. Whole-Genome Sequencing Using Oxford Nanopore Technologies (ONT)

ONT whole-genome sequencing data were generated as part of the 100 kGP long-read pilot (https://re-docs.genomicsengland.co.uk/ont/, last accessed on 10 July 2024). High molecular weight DNA for ONT sequencing was extracted from fresh/frozen human blood using the QIAGEN Gentra Puregene Blood Kit based on the ONT “High molecular weight gDNA extraction from whole rabbit blood—QIAGEN Gentra Puregene Blood Kit” protocol. Briefly, red blood cells (RBCs) from fresh or thawed (water bath at 37 °C) frozen blood were lysed using RBC Lysis solution (Gentra Puregene Blood Core Kit, QIAGEN, Hilden, Germany). The white blood cell (WBC) pellet was collected with brief Eppendorf centrifugation, and the RBC lysate supernatant was discarded. The WBC pellet was resuspended, lysed with Cell Lysis Solution (Gentra), and treated with RNase A Solution (Gentra). Proteins pellets were precipitated using Protein Precipitation Solution (Gentra) and centrifugation. The supernatant was collected, and DNA was precipitated using 100% isopropanol with gentle inversion and centrifugation. The DNA pellets were washed with 70% ethanol and eluted using DNA Hydration Solution (Gentra) for 2 h at 50 °C. The extracted DNA was stored at −20 °C.

For ONT sequencing, samples were thawed and quantified on a Qubit fluorometer (Q33226, Life Technologies, Paisley, UK) with the Qubit BR dsDNA assay (Q32853, Life Technologies, Paisley, UK), and manual volume checks were performed. The DNA size distributions were assessed at each relevant step using capillary pulsed-field electrophoresis with the FemtoPulse system (Agilent, Santa Clara, CA, USA, M5330AA and FP-1002-0275). Briefly, 3–9 μg gDNA was fragmented to a size of 20–50 kb with either the Megaruptor 3 (Diagenode, Seraing, Belgium, E07010001 and E07010003) or a gTUBE (Covaris, Brighton, UK, 520079) centrifuged at 1500 rcf. A subset of samples underwent an additional step to remove short DNA fragments (<10 kb) with the SRE kit (Circulomics, Baltimore, MA, USA, SS-100-101-01).

Sequencing libraries were generated from 1.8 μg or 1 μg of gDNA with either the SQK-LSK110 or SQK-LSK114 kit (ONT, Oxford, UK), respectively, according to the manufacturer’s recommendation with minor modifications. Briefly, samples were end-repaired by adding 2 μL NEBNext FFPE DNA Repair Mix (NEB, Ipswich, MA, USA, M6630) and 3 μL NEBNext Ultra II End Prep Enzyme Mix (NEB, E7546), incubated for 10 min at room temperature followed by 10 min at 65 °C, then cleaned up with 1 × AMPure XP beads (Beckman Coulter, Brea, CA, USA, A63880), and eluted in 60 μL of elution buffer. The end-repaired DNA was ligated with 5 μL Adapter Mix (ONT) using 8 μL NEBNext Quick T4 DNA ligase (NEB, E6056) at 21 °C for up to 1 h. The adapter-ligated DNA was cleaned by adding a 0.4× volume of AMPure XP beads. 

The libraries were quantified. Based on the average peak size of the samples determined after sample preparation on the Femto Pulse, 20 fmol of the obtained sequencing libraries was loaded onto a R9.4 flow cell (ONT), or 10 fmol of libraries was loaded onto R10 flow cells and sequenced on a PromethION48 (ONT). The libraries were stored overnight in the fridge. After 24 h, the runs were paused, and a DNAse treatment or nuclease flush (ONT, WSH-003) was performed. Then, 20 or 10 fmol of the libraries was re-loaded on the flow cells.

ONT data processing for SV detection followed the Genomics England “PromethION SV calling pipeline” (https://re-docs.genomicsengland.co.uk/PromethION%20SV%20calling%20pipeline%20GRCh38.docx, last accessed on 10 April 2024). Briefly, base calling was performed with Guppy high accuracy models, yielding merged fastq files that were mapped to GRCh38 with Minimap2. SVs were called using Sniffles or Sniffles2. Table 1 summarises versions of the tools used in the analysis pipeline for three separate batches of samples. Sniffles and Sniffles2 *de novo* calling performance was evaluated by checking the Sniffles/Sniffles2 outputs corresponding to each SV identified in the OGM reference callset. However, Case 7 was excluded since the parents were analysed with Sniffles in Batch 2, whereas the proband was analysed with Sniffles2 in Batch 3. Lastly, ONT data from two trios (Batch 3) were reanalysed using the Sniffles2 inbuilt population calling function. SV calls were deemed *de novo* when the read support for a variant call was >0 in the proband but = 0 in both parents. 

### 2.4. Optical Genome Mapping (OGM) Using the Bionano Platform

High molecular weight DNA for OGM was extracted from snap frozen blood using the “SP Blood & Cell Culture DNA Isolation Kit v2, product number: 80042” following the “Bionano Prep SP Frozen Human Blood DNA Isolation Protocol, 30246, revision F” protocol. Extracted DNA was processed for OGM using the “Direct Label and Stain (DLS) Kit, part number 80005” following the “Bionano Prep Direct Label and Stain (DLS) Protocol, 30206, revision G”. To collect OGM data, the DLS DNA was loaded into the Saphyr chip (version G1.2, two samples per chip) and mounted into the Saphyr machine, following the “Saphyr^®^ System User Guide, Document number 30143, revision C”. The maximum amount of data (1.5 Tb) was collected when possible. 

Subsequent computational analyses of the OGM data were performed on the Bionano Access platform (version 1.6.1) with the Bionano Solve software (version 3.6.1_11162020). Data from each OGM run were subjected to quality control (QC) to ensure run metrics met the recommended threshold according to the Bionano “Data Collection Guidelines, Document Number: 30173, Revision: E”. Quality control metrics for each sample analysis contributing to this work are provided in Table 2.

*De novo* assembly analysis was performed on each run. Rare SVs were extracted by setting both the “SV in less than this % of the control samples with the same enzyme” and “SV in less than this % of the control samples” to 0. Other filtering settings followed the recommended default values in the software and also in the Bionano document “Analysis Quick Start Annotated Rare Variant Structural Variant Calling, Rev A 30375”. The OGM reference SV set (*n* = 222) comprised a truth set of the rare OGM calls, verified by visual inspection of the raw reads/molecules with consensus from at least two of the three technologies. 

## 3. Results

### 3.1. ONT and OGM Run Qualities

ONT WGS was performed as part of the 100 kGP pilot ONT program and the Bionano OGM was undertaken at the MRC Weatherall Institute of Molecular Medicine, University of Oxford. Key metrics of the ONT and OGM runs are summarised in Table 2. For ONT data, while most samples achieved coverage comparable to that of the Illumina WGS at 30×, several cases, notably Case 3, had poor coverage that likely affected the SV detection performance. The N50 (average length of sequence) ranged from 16 kb to 49 kb, providing a good middle ground between Illumina WGS and Bionano OGM. Interestingly, no correlation was observed between the N50 and yield (coverage) in this dataset. For the OGM data, most runs achieved high quality at an ~90% map rate with >100× coverage. Case 3 had a poor map rate and therefore low effective coverage, which again is likely to have affected SV detection performance.

### 3.2. Bionano Benchmarks and Reference SV Set

From the nine trios, a total of 234 rare SV/CNV events were called using the Bionano Access software (Supplementary Table S1). These rare calls predominantly comprised deletions (DEL) and insertions (INS), including four likely duplicates (3 DEL and 1 INS), each identified in two different samples. There were a smaller number of CNVs (defined by the Bionano Access software as events > 500 kb), tandem duplications (DUP,) and complex events (CPX), the latter referring to multiple inversion and DUP calls from a local chromoanagenesis event on chr20 present in Case 8 that had been inherited from the unaffected mother (Table 3). This chr20 CPX event is described in detail by Pei (2024) [24]. 

For these 234 events, we manually examined the corresponding sequence data from Illumina and ONT in the Integrative Genomics Viewer (IGV) [25] to determine whether the SV could be independently verified and to confirm the SV type. Based on this analysis, 12 (2 CNVs, 3 DELs, 5 DUPs, and 2 INS) of 234 (5%) OGM calls were deemed false or uncertain based on the following reasons: (1) seven calls (*n* = 7) were near or at centromeric regions characterised by highly polymorphic genotypes for all three technologies; (2) three calls (*n* = 3) located at polymorphic regions of the genome (7q35, 9q13, and 16p12.3) without depth/coverage support from any of the three technologies; (3) two further calls (*n* = 2) were excluded comprising a challenging repeat locus on chrY, and a small DEL of ~500 bp near the detection limit.

For the remaining 222 supported calls, the original outputs from the Bionano Access SV caller were reclassified to be comparable with Illumina and ONT calls, as summarised in Table 3. As a result, the remaining CNV was reclassified as a DEL. From the chr20 CPX SV, several INV and DUP calls were made; these were reclassified into a single INS event to represent the most likely configuration. Notably, no true inversions (INV) or breakend events (BND) were called by Bionano Access in these nine cases. 

**Figure 1:**
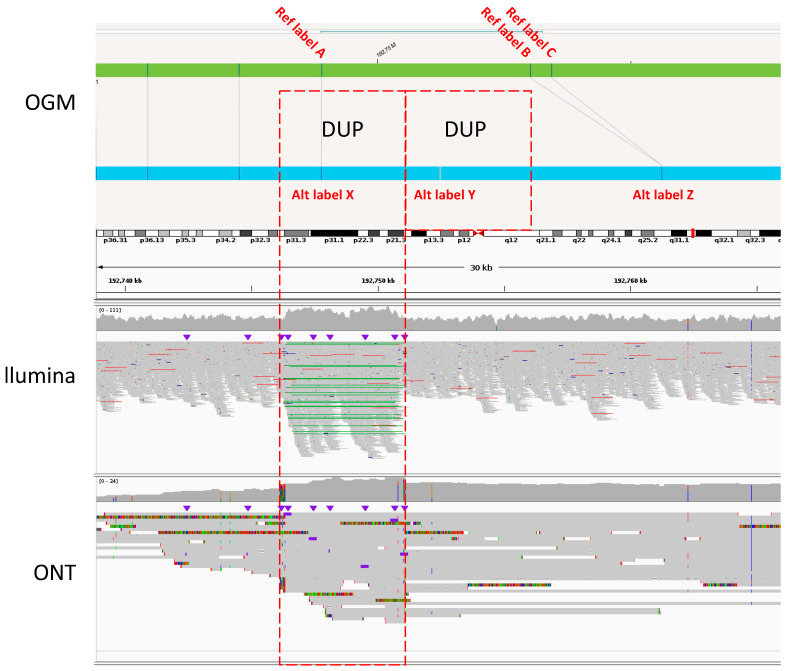
Example of DUP reclassification of an INS call made by the Bionano Access software. The figure shows the corresponding region of chromosome 1 analysed by Bionano OGM (**top**), Illumina SR-WGS (**middle**), and ONT LR-WGS (**bottom**) and aligned based on reference coordinates in hg38. In the OGM tracks, three labels of interest are highlighted for both the reference (Ref) map (labels A, B, C) and for the patient allele (Alt, labels X, Y, Z). Although an insertion of 4.7 kb of additional material is evident in the patient track, its nature is unclear because of the low resolution of OGM technology; this event was called as INS by the Bionano Access software. When assessed against the WGS data, evidence of a tandem DUP event is clearly evident in the Illumina track, where increased sequence coverage together with paired reads (green lines marking linked reads) establish its true identity. Note that increased sequence coverage is also evident in the ONT data (**bottom**), but the type of SV involved is not intuitively obvious. This SV was missed by Sniffles in Batch 1 data. Overall, it can be deduced that labels X and Y in the OGM track represent two copies of the same label due to the DUP, both corresponding to reference label A. Labels B and C map to label Z, possibly involving a dropout or merging of B and C.

The most substantial change was that 29% of the supported INS calls from the Bionano Access output were reclassified as DUPs, based on scrutiny of IGV reads from Illumina and/or ONT data (Table 3). The failure to distinguish DUP and INS events reliably was primarily attributable to the limitation imposed by the Bionano OGM’s labelling density, which typically averages around 6–7 kb separation between any two labels. Consequently, while Bionano OGM can detect an increased distance between the labels indicating a potential gain of genetic material, it cannot pinpoint the source of the extra material, if the gain is small in size and/or there is an inadequate number of informative labels. An example is illustrated in Figure 1, which shows an INS call by Bionano Access that was reclassified as a tandem DUP based on the read information from Illumina and ONT. 

**Figure 2:**
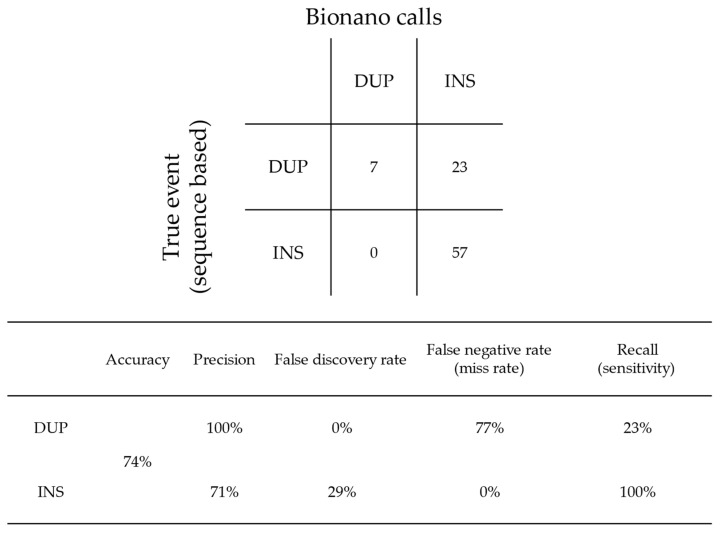
Error matrix and metrics for the INS and DUP events called by the Bionano *de novo* analysis pipeline. A true positive event was determined based on molecule and read evidence from at least two of the three technologies. Only reclassifications between DUP and INS were considered, i.e., CPX events reclassified into INSs were not considered in this matrix.

The occurrence of the DUP/INS misclassification as described above in the Bionano callset is summarised in Figure 2. Out of the 87 supported DUP/INS events (excluding the CPX event and false/uncertain calls), Bionano Access made 64 correct calls, yielding a 74% accuracy for DUP/INS calling. Specifically, when calling INS, Bionano Access is 71% precise, with 29% false discoveries (true DUPs falsely called as INS); when calling DUPs, Bionano Access is only 23% sensitive, as the remaining 77% DUPs are misclassified as INSs (false negative rate/miss rate).

In summary, the outcome of this OGM benchmarking exercise was that 222/234 Bionano Access calls were considered to be true events. Overall, this makes Bionano OGM ~95% precise at detecting rare SV events > ~500 bp, setting the Bionano OGM callset as a reliable reference for assessing the performance of Illumina and ONT WGS SV callers to detect the same set of rearrangements.

### 3.3. Illumina and ONT WGS Performance Evaluation

Illumina and ONT performances were evaluated using the reference SV set from the Bionano OGM analysis as discussed above. For the ONT data, Batch 1 and 2 were evaluated separately from Batch 3, owing to differences in the analysis pipelines and flow cells used. Major differences amongst the three batches are summarised in the Methods (see Table 1). There was no difference in the analysis pipeline for Illumina data between “batches” as all processes were carried out by Genomics England as part of the 100 kGP. The overall performance of Illumina and ONT is summarised in Table 4. 

One important observation was the minimal difference in the ONT performance purely based on read support between Batch 1 and 2 (R9 flow cells) and Batch 3 (R10 flow cells). This suggests that the newer R10 flow cells provided no particular benefit in SV detection. This finding is reasonable as the primary improvement for the R10 flow cells is reported to be the reduced base-calling error, which is mostly irrelevant for SV detection. Consequently, this suggests that the improved ONT performance in SV detection in Batch 3 is likely due to computational modifications, specifically the change to Sniffles2 as the SV caller for ONT (Table 4). 

As highlighted in Table 4, the original Sniffles SV caller underperformed substantially with only 48% sensitivity, missing nearly half of the OGM SVs in Batch 1 and 2. In comparison, Sniffles2 demonstrated improved performance with 84% sensitivity, exceeding even the performance of Illumina data with the union of three callers. This improvement in Sniffles2 may be attributed to the added repeat awareness and a coverage-adaptive filter when calling SVs [26]. This demonstrates that ONT LR sequencing has high potential to enhance SV calling compared to Illumina SR sequencing, if performed with the improved Sniffles2 SV caller. 

To gain a deeper understanding of the performance difference between ONT and Illumina, SV calls were stratified by the three detected SV types. Table 4 also summarises the sensitivity of ONT and Illumina for the three SV types. As shown, one of the most striking differences between ONT and Illumina is the INS sensitivity, where ONT–Sniffles2 is 52% more sensitive than Illumina with three callers combined. It is also interesting to highlight that, in data from the original Sniffles, the detection of all three types of SVs underperformed substantially, with only 55% of DELs called (expected to be easier to detect than DUP and INS events). This highlights the systematic underperformance of Sniffles in all SV types. Table 4 also illustrates the substantial improvements in calling all types of SV from the Sniffles2 caller. 

**Figure 3:**
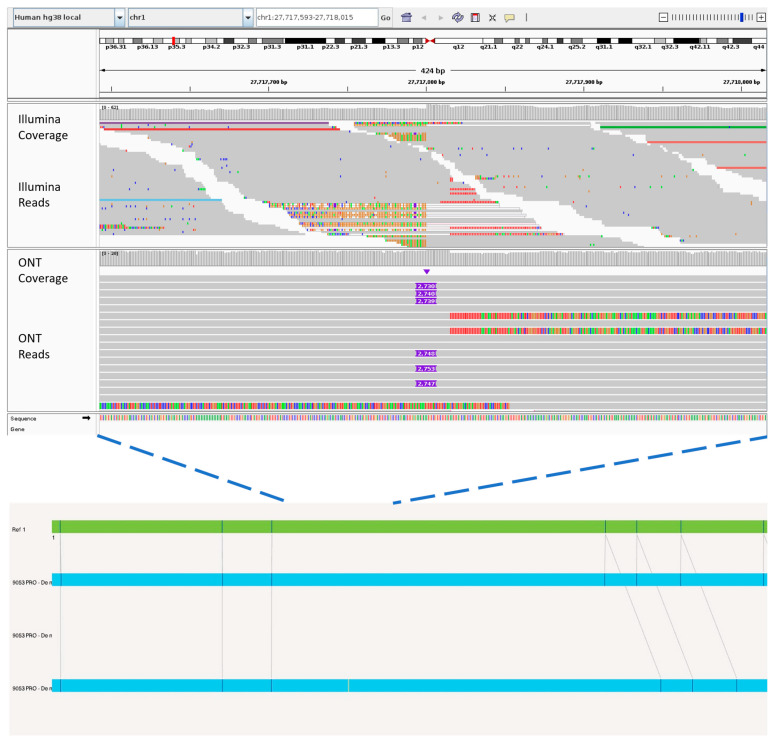
Example INS on chromosome 1 captured by ONT but not detected using Illumina WGS. Purple boxes in the ONT track (**middle panel**) clearly highlight the presence of the 2.7 kb INS, whereas Illumina short reads struggle to anchor properly owing to the size of the INS event (**top panel**). OGM was able to detect the INS (**bottom panel**, blue bars represent the two patient haplotypes), but the lack of labels prevent identification of the origin of the inserted sequences.

An example of the improvements made by Sniffles2 is the increased sensitivity of INS detection (74%). This is likely attributable to the long-read nature of ONT, providing additional anchoring capability for reads affected by INS events. As shown in Figure 3, an example of an INS of 2.7 kb can be clearly observed from ONT reads, as the ONT long reads can confidently span the entire INS sequences. In contrast, Illumina WGS cannot effectively anchor the short reads fully contained within the INS, resulting in failure to map these reads to the correct locus. Bionano OGM, on the other hand, detected the extra 2.7 kb sequence but could not pinpoint the source of this INS event due to the limit of labelling density. Analysis of the ONT reads further revealed that the inserted sequences consist of 2.5 kb of SVA_E and a short (GAGGGA)_n_ repeat, similar to the region of chr1:48,381,684–48,384,402 in the hg38 reference. 

### 3.4. ONT Performance—De Novo Calling

To evaluate the clinical utility of ONT sequencing in rare disease diagnosis, *de novo* calling was attempted for the ONT dataset, seeking clinically relevant SVs previously overlooked by both Illumina WGS and Bionano OGM. Therefore, the performance of *de novo* calling was evaluated. As shown in Table 5, the first two batches demonstrated poor performance in *de novo* calling, with 26% of the inherited SVs lacking the parental call, consequently being falsely classified as *de novo*. This poor performance of the initial ONT data rendered trio analysis uninformative due to the large portion of false *de novo* calls. In contrast, the Batch 3 data with Sniffles2 improved substantially, with only 8% inherited SVs falsely classified as *de novo*. This improvement is likely the result of the improved overall sensitivity of Sniffles2 as previously discussed and summarised in Table 4. 

To gain an understanding of the scale and feasibility of potential *de novo* analysis of ONT data in the future, a test analysis was carried out for the last two trios, namely, Cases 8 and 9 analysed in Batch 3. Overall, 268 (out of 38,260 total calls from Case 8) and 414 (out of 38,466 total calls from Case 9) *de novo* calls were made by Sniffles2 for Cases 8 and 9, respectively. These calls have at least one read support for the proband and none for both parents. Out of the *de novo* calls, 5 (Case 8) and 3 (Case 9) were shown to intersect with a coding region. Three of the coding calls were discarded as they affect highly polymorphic regions of the *MUC* genes. The remaining 5 candidate SVs are summarised in Table 6. After individual inspection in IGV, these 5 remaining candidates cannot be verified, based on the limited number of supporting reads. BND reads posed significant challenges to understand, as these are reads with partially soft-clipped sequences without reciprocal/corresponding reads from the other direction. The 5 candidate calls were also cross-examined using Illumina WGS reads and Bionano OGM molecules, which were all inconclusive. Illumina presented soft-clipped reads without mate reads; Bionano OGM showed no abnormal label or labelling distance. Overall, these calls may require further experimental work, such as targeted PCR and sequencing, to determine whether any are true positives.

## 4. Discussion

Short-read WGS, long-read WGS, and long-range OGM have different utilities and application in the clinic, particularly in identifying clinically relevant SVs in previously undiagnosed cases with rare genetic disorders. In this study, we conducted a comprehensive comparative analysis from data of nine trios analysed using three different genomics technologies: Illumina WGS, ONT WGS, and Bionano OGM. Using the OGM rare SV callset as the reference set, the performance of Illumina WGS and ONT was assessed relative to that of the Bionano OGM. Each of the SV calls was manually curated and examined using all three technologies, providing a thorough and detailed performance assessment in a validated set of rare SVs. Although other works have undertaken comparisons of different short- and long-range sequencing technologies with Bionano OGM for characterisation of SVs, these have either been applied to detection of somatically arising variants in cancers [27,28] or to the detailed characterisation of individual genomes, including common SVs [29,30]. We are not aware of any studies that have taken a Bionano-first approach to the characterisation of rare SVs identified in genomic data from multiple clinical samples, as described here.

Several key findings emerged from the analysis:

- Bionano OGM is highly precise, with 95% of rare SVs called as true events verified by molecules and/or reads supported from at least two technologies. However, Bionano OGM also misclassified 77% of tandem DUP as INS, owing to insufficient resolution for smaller DUPs, usually less than ~10 kb.
- Illumina WGS, with the union of three SV callers, successfully called 71% of events from the Bionano reference callset, with the most poorly detected SV type being INS at only 22% sensitivity.
- ONT performance is highly dependent on the variant caller. The original Sniffles performed poorly with a 48% sensitivity, and Sniffles2 demonstrated a significant improvement with 84% sensitivity.
- ONT performs much better at INS detection compared to Illumina.

These finding have important implications for detecting clinically relevant rare SVs. The high ~95% precision of Bionano OGM highlights its advantages in clinical settings, particularly where minimising false positive findings is crucial for the prompt and efficient delivery of clinically relevant results. Additionally, it is encouraging that Illumina and ONT WGS were able to call 71% (union of Canvas, Manta and Lumpy calls) and 84% (Sniffles2 caller) of the true Bionano SVs, respectively. This suggests that the OGM technology can provide added value, particularly in uncovering overlooked rare SVs from WGS.

INS detection remains one of the most significant challenges for Illumina WGS due to the limitation of short reads. Given that Illumina is currently the most common WGS approach in the clinical setting, there is likely a systematic underrepresentation of INS in clinically relevant SVs. Similar challenges with INS detection via Illumina have been reported in the literature, where Manta was benchmarked to have only ~8.6% sensitivity in real Illumina datasets [31]. Given that the truth dataset used by Kosugi et al. (2019) [31] consists of only PacBio SVs and existing DGV SVs from the samples, the reported sensitivity may not fully represent the diversity of INS in the human genome. In contrast, ONT–Sniffles2 likely offers the best solution for INS detection among the three technologies assessed, leveraging the advantage of long reads to achieve much higher sensitivity compared to Illumina, while maintaining sequence readability, which may be crucial to understand the true pathogenicity of INS events. 

A major limitation of the comparative analysis presented in this work is the lack of false negative assessment of the Bionano reference truth set. This limitation arises due to the challenges in identifying SVs missed by Bionano OGM. A complementary analysis to address this would involve deriving a truth set from the Illumina and/or ONT WGS data. However, given the project’s focus on manually curating an accurate set of clinically relevant rare SVs by scrutiny of IGV reads, it was impractical to undertake the same approach for the WGS data, primarily due to the substantial number (tens of thousands) of SV calls in each sample, the vast majority of which are likely to be false positive calls, in the Illumina and ONT datasets. In addition we did not attempt to evaluate systematically the efficacy of Bionano OGM in the interrogation of rare, complex SVs/translocations/inversions, as described by Dremsek et al. (2021) [32]. Nevertheless, one of the families analysed here (Case 8) is known to segregate a complex 4-break chr20 rearrangement (Section 3.2), which is likely incidental to the proband’s craniosynostosis.

The challenge of establishing truth datasets is frequently encountered when benchmarking different genomic technologies. Previous attempts have used simulated data by introducing artificial SVs into the test genome [31]. However, these efforts have revealed a near perfect SV calling performance in the simulated data, which does not align with assessments from real data. More recent efforts, such as the Genome in a Bottle (GIAB) Consortium, have aimed to compile a comprehensive truth set of SVs identified by a diverse range of genomic technologies, including Illumina, ONT, and PacBio [30]. Bionano OGM was also included, albeit primarily for verifying the SV length rather than for novel discoveries. Further comparative analyses of Bionano OGM performance against GIAB data could provide an interesting perspective. Another source of missed SV calls could stem from the inherent discrepancies in the hg38 reference genome. The recent Telomere-to-Telomere (T2T) assembly was undertaken to improve the accuracy of the reference genome [33]. However, it is important to note that many existing pipelines and databases for variant interpretation are yet to be fully migrated to the T2T assembly, making it less informative in the clinical setting. 

One further interesting observation is that there was only one true *de novo* SV event identified in the 234 rare Bionano callset (Supplementary Figure S1). This aligns with the consensus in the literature, stating that there is likely <1 *de novo* SV per generation [9,34]. However, within genomic regions accessible by sequencing technologies, there does seem to be a certain level of false positive *de novo* calls, as evidenced by the ONT–Sniffles false positive *de novo* calling rate. Similar observations were made from gnomAD SVs, where the apparent 3.0–7.4% *de novo* rate of SV calls were believed to be predominantly false negatives in the parents and/or false positive in the children [35]. In this study, Sniffles2 (ONT) performed well to reduce the false *de novo* calls by minimising false negative parental calls, while the (likely) false positive SVs in the children are yet to be experimentally assessed. Using the inbuilt trio-calling function in Sniffles2, 682 *de novo* calls were made from two ONT trios, amounting to ~350 *de novo* calls per trio. Given the consensus of expecting <1 *de novo* SV per generation, the number of *de novo* calls by Sniffles2 is highly overinflated with false positive calls. However, when compared to the apparent 3.0–7.4% *de novo* rate in gnomAD [35], Sniffles2–ONT performed better with an apparent *de novo* rate of only ~0.9% (682 *de novo* calls out of 76,826 total calls from two trios), suggesting either a lower false positive rate or a higher false negative rate for ONT compared to short reads in gnomAD; however, the latter (higher false negative for ONT) is less likely, as supported by the better sensitivity using ONT–Sniffles2 compared to Illumina as summarised in Table 4. 

It should be noted that given the expected low rate of *de novo* SVs, we did not regard it as cost-effective to perform routine OGM analysis in the parental samples. This was only undertaken in one trio (Case 8), and no *de novo* SVs were identified using the inbuilt trio analysis pipeline. These pieces of evidence all support the hypothesis that *de novo* SVs affecting coding regions or functionally critical non-coding regions are rare events in the human genome, emphasising their importance in clinical investigations. Of note, however the *bona fide de novo* SV call identified in this dataset (Supplementary Figure S1) is not believed to be causally linked to the phenotypes present in the respective proband.

With the improved Sniffles2 calling, *de novo* analysis was carried out to assess the feasibility of future SV analysis using ONT data in the clinical setting. This work focused on the latest two trios, yielding a manageable number of candidate calls as shown in Table 6. Following this robust *de novo* analysis, the entire cohort of 9 trios have been reanalysed using Sniffles2, yielding a total of 13 potential *de novo* calls affecting coding regions [24]. However, these calls could not be corroborated by supporting data from Illumina WGS and Bionano OGM. Additionally, the low number of reads, both reference and alternate in most calls (Table 6), suggests that these calls likely represent ONT-specific artefacts. Further PCR analysis would be necessary to support this conclusion. 

It is important to acknowledge that the decision to analyse Sniffles2 calls in the coding region only was due to time constraints, but it does mirror the time and resource limitation typically encountered in clinical settings. However, similarly to the WGS analysis, focusing on the non-coding regions to look for small-medium sized INS holds potential for identifying candidate pathogenic variants. This strategy may be particularly promising as these small INSs (50–500 bp) are most likely to be missed by both Illumina WGS and Bionano OGM. 

In conclusion, in this preliminary comparative analysis of three orthogonal datasets available for nine trios, we evaluated the performance of short- and long-range technologies in the clinical setting, with a focus on identifying rare/*de novo* SVs that are likely to have clinical implications. Bionano OGM stands out as a highly precise technology, despite its limitations in misclassifying small INS/DUP. Illumina WGS, with the union of three variant callers, demonstrated modest sensitivity (71%) measured against the Bionano OGM truth dataset. ONT WGS initially underperformed substantially, but the improved analysis pipeline with the enhanced Sniffles2 SV caller set out a strong foundation for future implementation of ONT as a viable way forward in clinical SV detection. The ONT-based approach will undoubtedly become more attractive as SV calling methods are further refined.

## Figures and Tables

**Table 1:** Major differences between the three batches in ONT analysis.

	BATCH 1	BATCH 2	BATCH 3
Number of proband samples	2	4	3
ONT Flow Cell	R9.4	R9.4	R10
Guppy	5.0.12 (on sequencer)	5.0.7 (on server)	6.4.6 (on server)
Minimap2 ^a^	2.20-r1061	2.24-r1122	2.24-r1122
Sniffles ^a^	1.0.11	1.0.11	2.0.6
Samtools ^a^	1.11	1.11	1.11
Tabix ^a^	1.9	1.9	1.9

^a^ Minimap2 was used for read alignment and mapping; Sniffles/Sniffles2 is the SV variant caller used specifically for ONT data; Samtools and Tabix were used for bam file processing, such as sorting and indexing.

**Table 2:** Run qualities and key metrics from ONT and OGM for all 9 trios.

				ONT Metrics							OGM Metrics					
Fam ID	Case ID	Sample	Batch	Estimated Bases Produced (Gb)	Estimated N50 (kb)	Estimated Coverage (×)	Total DNA (≥20 kb), Gb ^a^	Total DNA (≥150 kb), Gb ^a^	N50 (≥20 kb), kb ^b^	N50 (≥150 kb), kb ^b^	Average Label Density (≥150 kb),/100 kb ^c^	Map Rate (≥150 kb) ^d^	Effective Coverage (×) ^e^	Molecule Integrity Number ^f^	Positive Label Variance (PLV) ^g^	Negative Label Variance (NLV) ^h^
crs510	1	Proband	1	84.33	29.38	26	675.6	507.8	265.5	328.9	14.3	84.1%	132.08	0.08	2.2%	14.6%
		Mother	1	95.94	24.76	30										
		Father	1	90.91	27.75	28										
L16	2	Proband	1	156.92	26.62	49	944.3	768.1	336.4	409.9	17.9	81.5%	195.79	0.09	2.6%	9.0%
		Mother	1	104.41	24.59	33										
		Father	1	115.23	28	36										
M196	3	Proband	2	9.94	27.72	3	965.9	510.7	159.8	267.4	15.3	51.2%	77.88	0.12	3.0%	18.6%
		Mother	2	13.31	22.26	4										
		Father	2	37.93	19.73	12										
crs169	4	Proband	2	48.88	22.6	15	3189.4	1527.1	125.6	409.5	16.4	90.8%	432.94	0.07	2.9%	6.4%
		Mother	2	46.88	18.96	15										
		Father	2	55.98	34.31	17										
crs290	5	Proband	2	56.59	30.91	18	2163	1529	239.2	317.3	16.1	89.9%	417.7	0.1	2.4%	7.7%
		Mother	2	73.86	18.73	23										
		Father	2	47.56	24.24	15										
crs685	6	Proband	2	85.31	29.92	27	681.3	542	272.6	320.2	15.8	93.4%	157.1	0.08	2.4%	7.2%
		Mother	2	92.52	28.3	29										
		Father	2	82.81	35.21	26										
crs566	7	Proband	3	95.96	21.89	30	752.8	604.6	302.6	356.3	15.4	92.7%	173.54	0.07	4.3%	6.0%
		Mother	2	49.94	30.66	16										
		Father	2	35.71	32.48	11										
crs302	8	Proband	3	73.77	19.24	23	1855.6	1525.6	290.6	333.5	15.6	94.7%	450.36	0.07	2.3%	6.8%
		Mother	3	85.13	18.69	27	2027.1	1504.4	253.2	315.7	15.0	91.9%	430.46	0.07	2.1%	10.8%
		Father	3	85.45	19.07	27	2091.7	1464.2	231.8	308.6	15.8	91.7%	414.23	0.10	3.0%	7.1%
M44	9	Proband	3	53.16	18.53	17	1960.1	1508.4	257.2	308.6	15.5	92.9%	433.17	0.07	2.3%	7.7%
		Mother	3	77.93	20.56	24										
		Father	3	70.96	16.15	22										

^a^ Total amount of DNA from molecules that are 20 and 150 kb or longer, respectively; ^b^ Average length (N50) of DNA molecules that are 20 and 150 kb or longer, respectively; ^c^ Average number of labels per 100 kb for the molecules that are 150 kb or longer; ^d^ Percentage of molecules that are 150 kb or longer mapped to the reference; ^e^ Total amount of aligned DNA divided by the size of the reference genome times the map rate; ^f^ A measure of single molecule quality, where lower values are preferred and should be less than 20; ^g^ Percentage of labels absent in the reference; ^h^ Percentage of reference labels absent in molecules.

**Table 3:** SV types and normalisation into DEL, tandem DUP, and INS events from 234 Bionano Access SV calls.

	CNV	CPX	DEL	Tandem DUP	INS	False/Uncertain
OGM Calls	3	1	136	12	82	-
Reclassified/Normalised	0	0	134	30	58	12

**Table 4:** The overall and per type SV detection performance of ONT and Illumina compared to Bionano OGM.

Data Batches	Technology— SV Detection Method	Overall Performance			Performance by SV Type			
		TP	FN	Sensitivity	SV Type	TP	FN	Sensitivity
Batch 1, 2, and 3	Illumina—M + C + L ^a^				DEL	115	19	86%
		157	65	71%	DUP	29	1	97%
					INS	13	45	22%
Batch 1 and 2 R9 Flow Cells	ONT—Read Support ^b^				DEL	93	1	99%
		148	6	96%	DUP	23	2	92%
					INS	32	3	91%
	ONT—Sniffles				DEL	52	42	55%
		74	80	48%	DUP	9	16	36%
					INS	13	22	37%
Batch 3 R10 Flow Cells	ONT—Read Support				DEL	38	2	95%
		63	5	93%	DUP	5	0	100%
					INS	20	3	87%
	ONT—Sniffles2				DEL	36	4	90%
		57	11	84%	DUP	4	1	80%
					INS	17	6	74%

^a^ Illumina SV calls consist of a union of three variant callers, namely, Manta (M), Canvas (C), and Lumpy (L). ^b^ Owing to the suboptimal performance of Sniffles in Batch 1 and 2, ONT read support was manually evaluated to determine if the reduced performance originated experimentally or computationally. Unpaired breakends (BNDs) were considered as negative calls.

**Table 5:** *De novo* calling performance improved in Batch 3 using Sniffles2 compared to the first two batches using Sniffles.

		FP *de novo* ^a^ (Parental Call Missing)	TN *de novo* ^b^ (True Inherited Calls)	False Positive Rate (Probability of False *de novo* Calls)
Batches 1 and 2	ONT–Sniffles	19	55	26%
Batch 3	ONT–Sniffles2	3	36	8%

^a^ False positive (FP) *de novo* calls are SVs inherited from a parent, where the parental SV was not called by Sniffles or Sniffles2. ^b^ True negative (TN) *de novo* calls are inherited SVs called correctly in both the proband and the parent. The false positive rate was calculated as FP/(FP + TN). One TP *de novo* variant was called in Batch 3 (Supplementary Figure S1) and is not included in this table. Case 7 was excluded from this analysis as the parents were analysed with Sniffles in Batch 2, while the proband was analysed with Sniffles2 in Batch 3.

**Table 6:** Five coding, potentially *de novo* candidates from the ONT–Sniffles2 analysis.

Case	Chromosome	Position	SV Length	SV Type	Sniffles2-Ref Reads	Sniffles2-Alt Reads
8	chr15	43,572,861	.	BND	2	2
8	chr19	1,993,025	70,128	INS	1	11
9	chr12	52,517,168	.	BND	2	2
9	chr16	69,961,666	6377	INS	5	3
9	chr4	39,308,656	.	BND	4	2

“Ref reads” are reads supporting the reference call, while “Alt reads” are reads supporting the variant call made by Sniffles2.

## Data Availability

All Illumina and ONT sequence data are available in the National Genomic Research Library to approved researchers.

## Supplementary Materials

The following supporting information can be downloaded at: https://www.mdpi.com/article/10.3390/genes15070925/s1, Figure S1: A *bona fide de novo* SV identified in the M44 proband. Table S1: Comprehensive list of 234 SV calls identified by Bionano OGM with annotations from downstream analyses, including SV call normalisation, comparative analysis with ONT and Illumina WGS, and *de novo* calling analysis from ONT.
