# Supplementary material for: A Comparison of Structural Variant Calling from Short-Read and Nanopore-Based Whole-Genome Sequencing Using Optical Genome Mapping as a Benchmark
Source: Genes (Basel). 2024 Jul 16;15(7):925. doi: 10.3390/genes15070925 (PMC11276380; doi:10.3390/genes15070925)
Supplement: Supplementary file 1 [file genes-15-00925-s001.zip › Supplementary Figure S1.pdf]

**Supplementary Material: Comparison of Structural Variant Calling from Short-Read and Nanopore-Based Whole-Genome Sequencing Using Optical Genome Mapping as a Benchmark, by Yang Pei *et al*.**

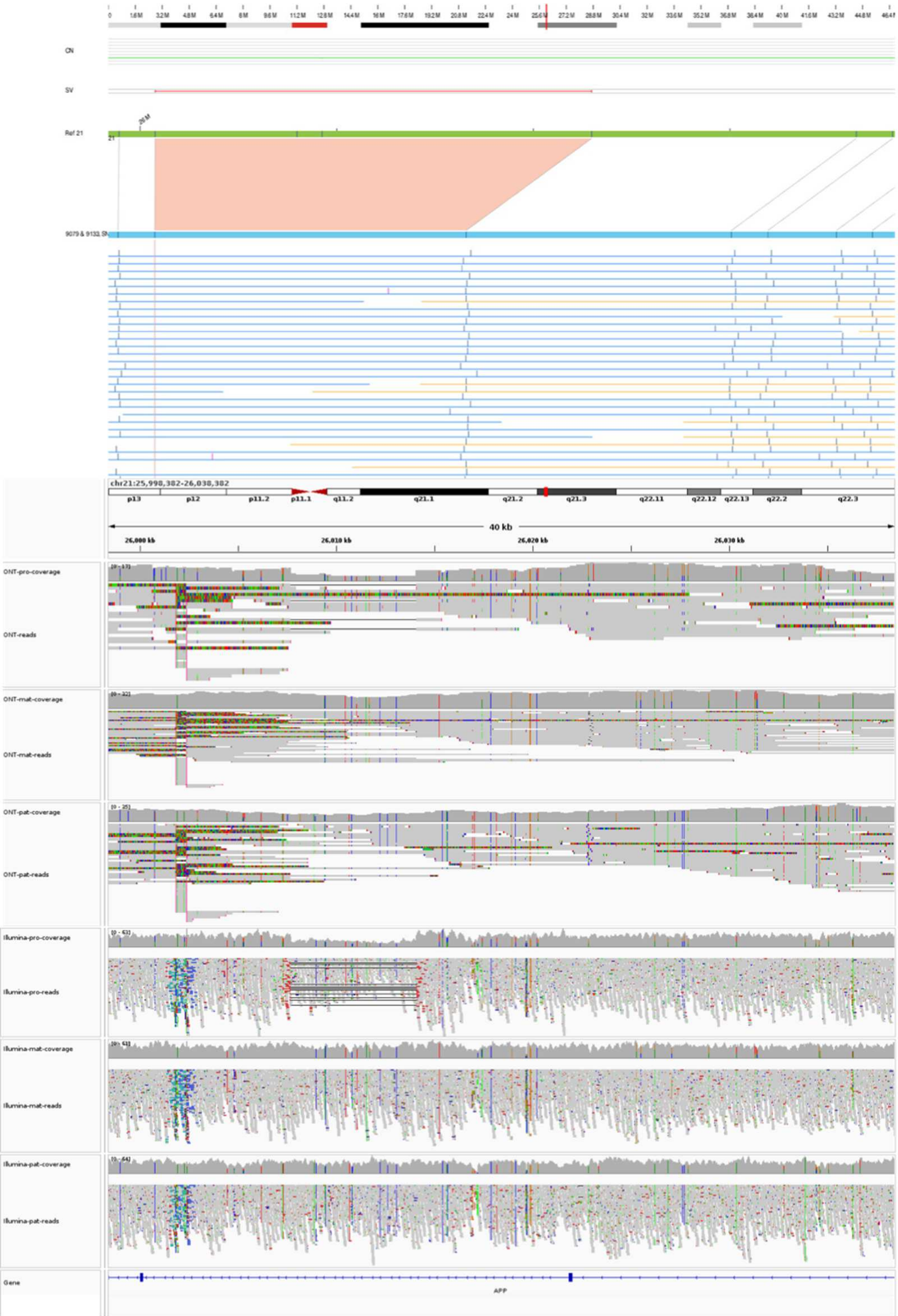


**Supplementary Figure S1.** A *bona fide de novo* SV identified in the M44 proband. This ~6.4 kb deletion locates within the 6th intron of *APP*. The deletion was successfully called in both the Illumina (Manta only) and ONT (Sniffles2 in Batch 3) analyses. Upper panel shows the Bionano OGM overall assembly for the proband data in the ~40 kb region with multiple supporting molecules (individual blue and orange lines) aligned under the map. Lower panel shows the IGV plot for 6 data tracks ordered as follows: ONT data for the proband, mother, and father; Illumina data for the proband, mother, and father. The *de novo* deletion is evident from the reduced labeling distance between two adjacent labels in the OGM molecules, and the drop in coverage and abnormal split-reads from the Illumina and ONT sequencing.
